# Deregulated HOXB7 Expression Predicts Poor Prognosis of Patients with Esophageal Squamous Cell Carcinoma and Regulates Cancer Cell Proliferation In Vitro and In Vivo

**DOI:** 10.1371/journal.pone.0130551

**Published:** 2015-06-15

**Authors:** Hui Li, Lu-Yan Shen, Wan-Pu Yan, Bin Dong, Xiao-Zheng Kang, Liang Dai, Yong-Bo Yang, Hao Fu, He-Li Yang, Hai-Tao Zhou, Chuan Huang, Zhen Liang, Hong-Chao Xiong, Ke-Neng Chen

**Affiliations:** 1 Key Laboratory of Carcinogenesis and Translational Research (Ministry of Education), Department of Thoracic Surgery I, Peking University Cancer Hospital & Institute, Beijing, People’s Republic of China; 2 Key Laboratory of Carcinogenesis and Translational Research (Ministry of Education), Department of Pathology, Peking University Cancer Hospital & Institute, Beijing, People’s Republic of China; The University of Hong Kong, HONG KONG

## Abstract

**Background:**

We observed abnormal HOXB7 expression in esophageal squamous cell carcinoma (ESCC) previously. This study was to evaluate the prognostic significance of HOXB7 and reveal the potential mechanism.

**Methods:**

Immunohistochemistry was used to confirm the abnormal expression of HOXB7 in ESCC. The prognostic significance of HOXB7 expression was analyzed in two independent cohorts. RNAi was used to establish two stable HOXB7-knockdown cell strains. CCK8 assay, cell growth curve assay, colony formation assay, flow cycle analysis and tumorigenicity assay in nude mice were employed to investigate the effect of HOXB7 on proliferation in vitro and in vivo.

**Results:**

Immunohistochemistry confirmed the abnormal expression of HOXB7 in ESCC compared with paracancerous mucosa (18/23 vs. 9/23, p=0.039). HOXB7 expression was positively correlated with the T stage, lymph node metastasis and TNM stage. The median survival of patients with high HOXB7 expression was significantly shorter than that with low expression (45 months vs. 137 months, p = 0.007 for cohort 1; 19 months vs. 34 months, p = 0.001 for cohort 2). Multivariate survival analysis showed that HOXB7 expression was another independent prognostic factor (HR [95% CI] = 0.573 [0.341–0.963], p = 0.036 for cohort 1; HR [95%CI] = 0.543 [0.350–0.844], p = 0.024 for cohort 2). Experiments in vitro and in vivo showed that after knockdown of HOXB7, the proliferation rate dropped, growth rate descended, colony-formation ability reduced, G1-phase arrest occurred and the tumorigenicity reduced remarkably.

**Conclusions:**

HOXB7 could promote cancer cell proliferation and might be an independent prognostic factor for patients with ESCC.

## Introduction

Currently, esophageal cancer is one of the most common malignancies worldwide, and esophageal squamous cell carcinoma (ESCC) is the most common pathological type in the Chinese population [1, 2]. Although the surgery-dominated multidisciplinary treatment has significantly advanced in the past two decades with respect to the diagnosis and treatment of ESCC, and the outcome of treatment has been improved, the long-term survival is still unsatisfactory [3]. One of the effective measures to improve overall survival involves the identification of clinical biomarkers with prognostic significance to guide treatment. HOX genes are a group of genes regulating embryonic development. The family, with 39 members, is divided into four clusters, namely, A, B, C, and D, which are located on four different chromosomes [4, 5]. HOX genes encode a family of transcription factors, and their abnormal expression is often associated with diseases, thus attracting increasing attention in cancer research [6, 7]. Previous studies in developmental biology have shown that HOXB7 is normally expressed in the development of human esophagus [8]. Among the 39 HOX genes examined in our previous study, eight were expressed only in the malignant tissue from ESCC patients, but not in the paired adjacent normal tissues, including HOXB7 with 58.3% positive expression rate [9]. Therefore, we hypothesized that HOXB7 played a specific role in the occurrence and development of ESCC and might be a useful molecular marker for predicting prognosis and even guiding treatment. To the best of our knowledge, there are rare studies on HOXB7 expression and prognosis in ESCC involving a large sample size, and no in vivo and in vitro studies discussing the underlying mechanism. Therefore, the present study aimed to investigate the prognostic significance and functional mechanism of HOXB7 in ESCC tissues.

## Materials and Methods

### Ethics Statement

All research involving human participants was approved by the Ethics Committees of Beijing Cancer Hospital, China, and conducted in accordance with the Declaration of Helsinki. Written informed consents have been obtained from all participants.

### Patients and tissue samples

To validate the high expression of HOXB7 in ESCC tissues reported in our previous studies [9], 23 ESCC patients were recruited and the paraffin-embedded tumor and paired noncancerous tissue sections were retrieved.

To investigate the association of HOXB7 protein expression with prognosis of patients with ESCC, two independent study cohorts were included:

#### The cohort I

Our research group established a prospective database for esophageal cancer since Jan. 2000 and 1249 cases with esophagectomy performed by a single-surgeon team (Dr. KN Chen) have been consecutively collected. In this study, the inclusion criteria was as follows: the patients who were pathologically diagnosed with ESCC and underwent radical esophagectomy without induction therapy before surgery between Jan. 2000 and Dec. 2011. A total of 177 cases met the criteria (Table 1). The 7th edition of TNM staging system (AJCC and UICC, 2009) was used to classify patients.

**Table 1:** Association between HOXB7 expression and clinical characteristics of patients with ESCC in the cohort I (n = 177) and cohort II (n = 103).

Variables	HOXB7 expression in cohort I			HOXB7 expression in cohort II		
	High levelNo.(%)	Low level No.(%)	P value	High level No.(%)	Low level No.(%)	P value
**Age (y)**						
** ≤60**	**47 (73.4)**	**17 (26.6)**	**0.460**	**24 (45.5)**	**20 (54.5)**	**0.404**
** >60**	**77 (68.1)**	**36 (31.9)**		**37 (62.7)**	**22 (37.3)**	
**Gender**						
** Male**	**93 (72.7)**	**35 (27.3)**	**0.222**	**43 (59.7)**	**29 (40.3)**	**0.875**
** Female**	**31 (63.3)**	**18(36.7)**		**18(58.1)**	**13 (41.9)**	
**Tumor location**						
** Cervical**	**5 (71.4)**	**2 (28.6)**	**0.709**	**0(0)**	**0(0)**	**0.890**
** Upper**	**20 (64.5)**	**11 (35.5)**		**6 (60.0)**	**4 (40.0)**	
** Middle**	**58 (74.4)**	**20 (25.6)**		**38 (57.6)**	**28 (42.4)**	
** Lower**	**41 (67.2)**	**20 (32.8)**		**17 (63.0)**	**10 (37.0)**	
**Tumor cell differentiation**						
** Well**	**19 (61.3)**	**12(38.7)**	**0.370**	**29 (64.4)**	**16 (35.6)**	**0.599**
** Moderate**	**59 (69.4)**	**26 (30.6)**		**17 (53.1)**	**15 (46.9)**	
** Poor**	**46 (75.4)**	**15 (24.6)**		**15 (57.7)**	**11 (42.3)**	
**Tumor invasion (T)**						
** Tis&T1&T2**	**49 (62.0)**	**30 (38.0)**	**0.036**	**6(25.0)**	**18 (75.0)**	**0.000**
** T3&T4**	**75 (76.5)**	**23 (23.5)**		**55 (69.6)**	**24(30.4)**	
**Lymph nodes metastasis (N)**						
** N-**	**79 (65.3)**	**42 (34.7)**	**0.042**	**25 (48.1)**	**27 (51.9)**	**0.006**
** N+**	**45 (80.4)**	**11 (19.6)**		**36 (70.6)**	**15 (29.4)**	
**TNM stage**						
** 0**	**4 (80.0)**	**1 (20.0)**	**0.034**	**0(0)**	**0(0)**	**0.001**
** I**	**16 (51.6)**	**15 (48.4)**		**1 (25.0)**	**3 (75.0)**	
** II**	**57 (68.7)**	**26 (31.3)**		**22 (43.1)**	**29 (56.9)**	
** III**	**47 (81.0)**	**11 (19.0)**		**36 (78.3)**	**10 (21.7)**	
** IV**	**0(0)**	**0(0)**		**2 (100)**	**0 (0)**	

#### The cohort II

The cohort II included 103 patients who were pathologically diagnosed with ESCC and underwent radical esophagectomy by multiple surgeons without induction therapy before our prospective database established from 1996 to 2002 (Table 1). The data was retrospectively collected according to patient's medical records without prospective data. The TNM staging system of UICC, 1987 was used to evaluate patients.

### Follow-up methods

The follow-up data of cohort I was collected from our prospective database. After surgery, outpatient follow-up visits were conducted once in every 3months in the first 2 years, once in every 6 months from 2 to 5 year, and once in every year after 5 years. The overall survival was defined as the time from the date of surgery to death or the last follow-up. The last follow-up checkpoint was Aug. 2013. The total follow-up rate for our database was 96.1%: 80.2% outpatient visits and 15.9% telephone or letter follow-up. The telephone or letter follow-up mainly involved those patients without visiting to outpatient clinic on schedule. Outpatient follow-ups involved recording of symptoms and multiple examinations including CT, upper gastrointestinal imaging, abdominal and bilateral supraclavicular ultrasound, and gastroscopy, if necessary. After 2010, some patients underwent positron emission tomography CT (PET-CT) examinations.

The follow-up data of cohort 2 were collected from the review of patient's medical records. The last follow-up time was Feb, 2008 and follow-up rate was 80%.

### Cell cultures

The human ESCC cell lines EC109 and KYSE150 were obtained from Cell Bank of Chinese Academy of Medical Sciences, Beijing, China. EC109 was cultured in DMEM medium (Gibco) supplemented with 10% FBS (Gbico) at 37°C in a 5% CO2 air atmosphere. KYSE150 was cultured in RPMI 1640 medium (Gibco) with 10% FBS (Gbico) at 37°C in a 5% CO2 air atmosphere.

### Vectors construction and lentiviral infection

For down-regulation of HOXB7, four short hairpin (shRNA) sequences (GCCGAGTTCCTTCAACATGCA/CGAGAGTAACTTCCGGATCTA/GCCTCACGGAAAGACAGATCA/CGAGAGTAACTTCCGGATCTA/GCCTCACGGAAAGACAGATCA/ACCTGTTCTGTAGCTTTCTGG) were cloned into pGLV-h1-GFP-puro. Scrambled sequence (ACTACCGTTGTTATAGGTG) were used as controls. Lentiviral vector construction and packaging was performed by GenePharma Company (Shanghai, China). Cells were infected with 50ul filtered lentivirus supernatant (1 × 10^9^ TU/ml) in each well of 24-well plate with the presence of 8 μg/ml polybrene (Millipore). Stable transfected cell strains expressing shHOXB7 were selected with 2 ug/ml puromycin.

### RNA extraction, RT-PCR and real-time PCR

Total RNA of cells was extracted by TRIzol (Invitrogen, Carlsbad, California). RNA was reverse transcripted to cDNAs by RT-PCR (Fermentas Life Science). Quantitative Realtime PCR was performed using SYBR Green Realtime PCR Maser Mix (Applied Biosystems) to detect mRNA expression levels of target genes. GAPDH (glyceraldehyde-3-phosphate) was used as endogenic control. The sequence of the primers used are as follows: for HOXB7, forward 5'-TATGGGCTCGAGCCGAGTT- 3', reverse 5'-GGCCTCGTTTGCGGTCAGT -3'; for GAPDH, forward 5'-TGCACCACCAACTGCTTAGC -3', reverse 5'- GGCATGGACTGTGGTCATGAG -3’. All assays were carried out in triplicate under 7500 real-time PCR system (Applied Biosystems) and repeated three times according to the manufacturer’s protocol. Evaluation of relative expression was calculated by comparative CT (threshold cycle) method. 2^-ΔΔCt^ referred to the fold of the mRNA expression of the target gene compared to GAPDH expression in the same sample.

### Western blotting

Total cell extracts were prepared in 1×SDS loading buffer, separated by SDS-PAGE, blotted into PVDF membranes, then immunoreacted with mouse monoclonal anti-HOXB7 antibody (Abnova) as primary antibody. Goat anti-mouse horseradish peroxidase—conjugated IgG was used as a secondary antibody. Immunoreactivity was detected with an enhanced chemiluminescence reaction kit (GE Healthcare). As a loading control, glyceraldehyde- 3-phosphate dehydrogenase (GAPDH) was detected using a mouse monoclonal antibody (Abcam).

### Immunohistochemistry

After routine deparaffinization and hydration, tissue sections were treated with 3% hydrogen peroxide and then heated in citrate solution (pH = 6.0) for antigen retrieval. The HOXB7 antigen-antibody reaction took place overnight at 4°C after goat serum blocking. The mouse monoclonal anti-HOXB7 antibody (Abnova) was used at 1 μg/ml (1:1000), and goat anti-mouse biotin-conjugated IgG was used as secondary antibody. The immunohistochemical signals were scored by two independent observers. The scores were calculated as the number of stained cells divided by the total number of cancer cells. Five representative high-power fields (X400) per slide were calculated and the results were averaged. Unequivocal staining of the nucleus in >25% of cancer cells was considered high expression.

### CCK8 assay and cell growth curve

The cell proliferation was determined by Cell Counting Kit-8 staining (Dojinodo, Shanghai, China) according to the manufacturer's instructions. Cells were seeded on 96-well plates at initial density of 5×10^3^ cells/well. At each time point, the cells were stained with 10μl CCK8 dye in 90μl culture medium for 2 h at 37°C. The absorbance was measured at 450 nm, with 650 nm as the reference wavelength. The results are confirmed by manual cell counts. Cells were seeded on 24-well plates at initial density of 5×10^3^ cells/well and collected at different time and counted using a hematocytometer. All experiments were performed in triplicates.

### Colony formation assay

Cells were trypsinized and plated in 6-well plates (300–500 cells/well) and cultured for 3 weeks. The colonies were stained with Giemsa stain for 30min after fixation with 4% paraformaldehyde for 15 minutes. Three independent experiments were performed.

### Cell cycle analysis

Cell cycle distribution was examined by flow cytometry. 1x10^6^ cells were collected and fixed with 70% cold ethanol. After fixation, the PI-staining solution with RNase A (BD Biosciences) was added. After 30 min of incubation, stained samples were run on the FACScan cytometry (BD Biosciences), and data were analyzed using FlowJo software (Tree Star).

### Tumorigenesis in nude mice

Animal experiments were approved by the Ethics Committees of Beijing Cancer Hospital, China, and conducted in accordance with the Guide for Care and Use of Laboratory Animals. Mice were maintained under the care of the Laboratory Animal Unit of Beijing Cancer Hospital, China. Stable shRNA transfected cells (1 × 10^6^) in 100 μL serum-free DMEM/RPMI 1640 were injected s.c. into the right flank of 4 to 6 weeks old female Balb/C athymic nude mice. All mice were housed and maintained under specific pathogen-free conditions. Xenograft tumors were generated and the mice were sacrificed by cervical dislocation within 4 weeks. Tumors were excised, measured by a slide caliper and weighted by an electronic analytical balance. Tumor volumes were calculated using the following formula: tumor volume = [(length) × (width) × (width)] / 2. All experiments were done in accordance with institutional standard guidelines of Peking University School of Oncology for animal experiments.

### Statistical analysis

All statistical analyses were performed using the SPSS 19.0 statistical software package. All in vitro experiments were performed at least thrice in triplicates. Comparisons between groups for statistical significance were performed with a 2-tailed unpaired Student’s t test. Bars and error bars on the graphs as well as data in the text represent the mean ± SD. Comparisons between the cancer and paired normal tissues were tested using Wilcoxon signed rank test. The relationships between HOXB7 expression and clinicopathologic characteristics were tested using Chi-square test. Survival curves were plotted by Kaplan—Meier method and compared by log rank test. The Cox proportional hazards model with a stepwise procedure was used for multivariate analysis. P < 0.05 was considered statistically significant.

## Results

### The expression of HOXB7 was upregulated in ESCC compared with paired noncancerous tissues

**Fig 1:**
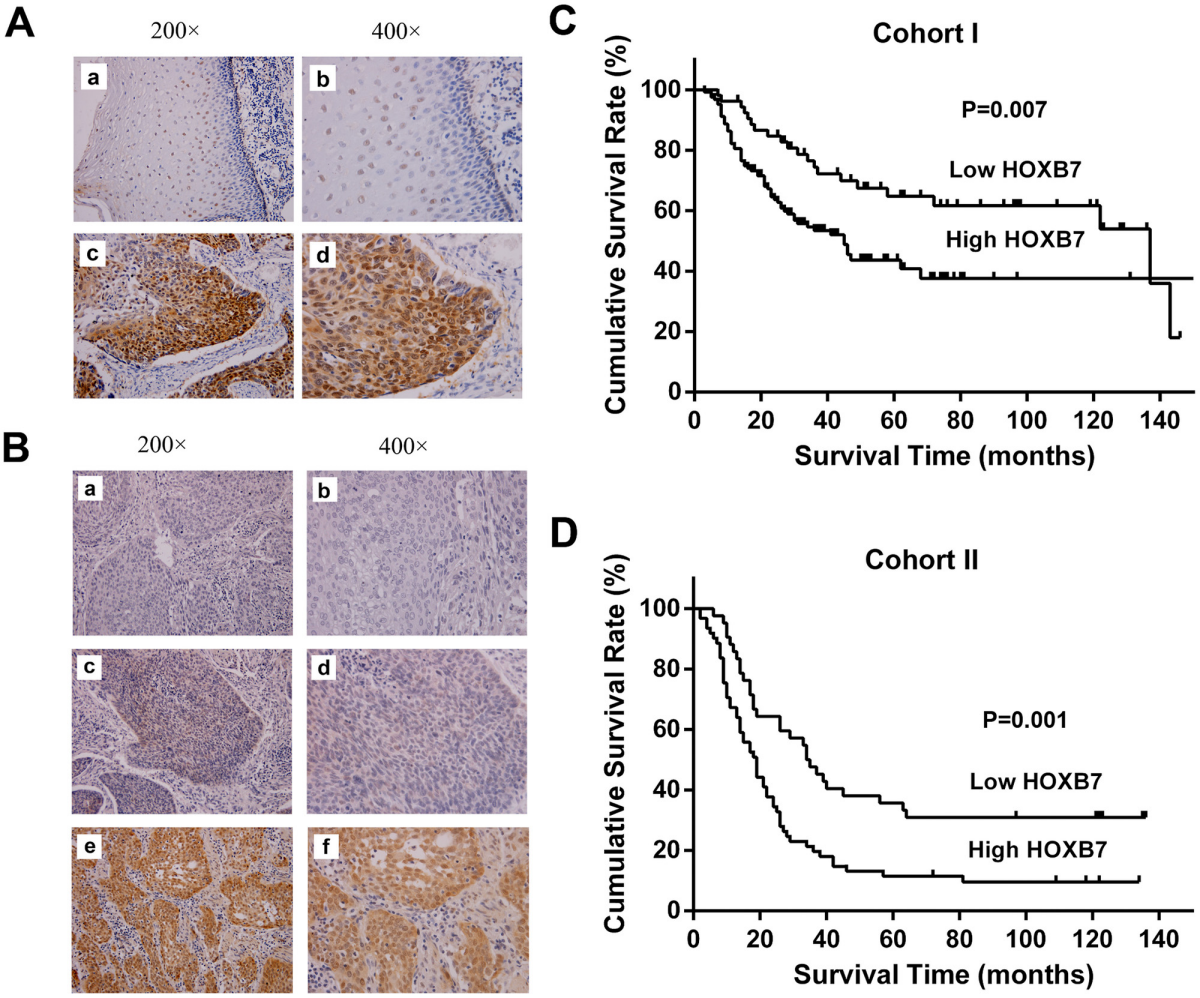
Immunohistochemical detection of HOXB7 in ESCC specimens and Kaplan-Meier survival curve for ESCC patients. (A) Representative sample of paired normal tissues (a, b) and ESCC (c, d). In the normal esophageal epithelial, HOXB7 expression was mainly limited to the nucleus of the epithelial cells located in the basal and suprabasal layers, whereas in the ESCC, HOXB7-positive cells were broadly observed in the tumor (Case no. 53865). (B) Figure a and b, negative control, with primary antibody replaced by PBS (Case no. 40146). Figure c and d, low expression of HOXB7 in ESCC (Case no. 42873). Figure e and f, high expression of HOXB7 in ESCC (Case no. 36840). (C) Kaplan-Meier survival curve for 177 patients in the cohort I. The median survival time was 45 months for high expression patients, which was significantly shorter than the 137 months for low expression patients (P = 0.007). (D) Kaplan-Meier survival curve for 103 patients in the cohort II. The median survival time was 19 months for high expression patients, which was significantly shorter than the 34 months for low expression patients (P = 0.001).

We had previously reported 8 of 39 HOX genes, including HOXB7, abnormally expressed in ESCC tissues but not in noncancerous tissues using reverse transcription-PCR [9]. To further elucidate the expression of HOXB7 protein in ESCC, immunohistochemistry (IHC) stain was performed in tumors and paired noncancerous tissues from ESCC patients. It was noted that HOXB7 protein was mainly localized in the nucleus of the normal esophageal epithelial cells in the basal and suprabasal layers, whereas in the tumors, HOXB7-positive cells distributed widely. Comparative analysis indicated that HOXB7 was significantly upregulated in 23 examined tumor samples (18/23) compared with adjacent noncancerous tissues (9/23) (Fig 1A, P = 0.039).

### HOXB7 protein expression was associated with clinical characteristics and overall survival

In cohort I, high expression of HOXB7 protein was observed in 124 of 177 (70.1%) tumor samples (Fig 1B). Chi-square test showed that the expression levels of HOXB7 protein significantly correlated with TNM stage (P = 0.034), T stage (P = 0.036) and lymph node metastasis (Table 1, P = 0.042). Univariate analysis indicated that patients who had low HOXB7 expression levels lived much longer (Fig 1C, P = 0.007). The median survival time was 45 months for patients with high expression, which was significantly shorter than the 137 months for patients with low expression (Table 2). Multivariate survival analysis confirmed that the HOXB7 expression and TNM stage were two independent prognostic factors for overall survival in patients with ESCC (Table 3, HR [95% CI] = 0.573[0.341–0.963], p = 0.036 for HOXB7 low expression).

**Table 2:** Association between HOXB7 expression and survival time of patients with ESCC.

Variables	No. (%)	Median survival time (95%CI)	P value
**Cohort I**			
**HOXB7(<25%)**	**53(29.9%)**	**137(57.511–216.489)**	**0.007**
**HOXB7(≥25%)**	**124(70.1%)**	**45(31.877–58.123)**	
**Cohort II**			
**HOXB7(<25%)**	**42(40.8%)**	**34(23.415–44.585)**	**0.001**
**HOXB7(≥25%)**	**61(59.2%)**	**19(14.655–23.345)**	

**Table 3:** Independent predictors of survival time in multivariate analysis.

Variables	Hazard ratio (95% confidence interval)	P value
**Cohort I**		
** TNM stage**		**0.000**
** Stage 0 vs. stage III**	**0.000 (0.000–9.66E237)**	**0.964**
** Stage I vs. stage III**	**0.355 (0.13–0.687)**	**0.002**
** Stage II vs. stage III**	**0.324 (0.19–0.527)**	**0.000**
** HOXB7 expression**		**0.036**
** Low vs. High**	**0.573 (0.341–0.963)**	**0.036**
**Cohort II**		
** TNM stage**		**0.043**
** Stage I vs. stage IV**	**0.280 (0.038–2.063)**	**0.288**
** Stage II vs. stage IV**	**0.565 (0.135–2.363)**	**0.446**
** Stage III vs. stage IV**	**0.997 (0.240–4.137)**	**0.933**
** HOXB7 expression**		**0.024**
** Low vs. High**	**0.543 (0.350–0.844)**	**0.024**

In cohort II, the high expression of HOXB7 protein was detected in 61 of 103 (59.2%) cases of tumor tissue samples (Fig 1B). Chi-square test showed that the expression levels of HOXB7 protein significantly correlated with TNM stage (P = 0.001), T stage (P = 0.000) and lymph node metastasis (Table 1, P = 0.006). Univariate analysis using the log rank test showed that patients with low HOXB7 expression levels had a longer median survival time than that with high HOXB7 expression (34 mons vs. 19 mons, P = 0.001) (Fig 1D,Table 2). Multivariate survival analysis indicated that the HOXB7 expression level and TNM stage were two independent prognostic factors for overall survival in patients with ESCC (Table 3, HR[95%CI] = 0.543[0.350–0.844], p = 0.024 for HOXB7 low expression).

### Down-regulation of HOXB7 protein decreased cell proliferation in vitro

**Fig 2:**
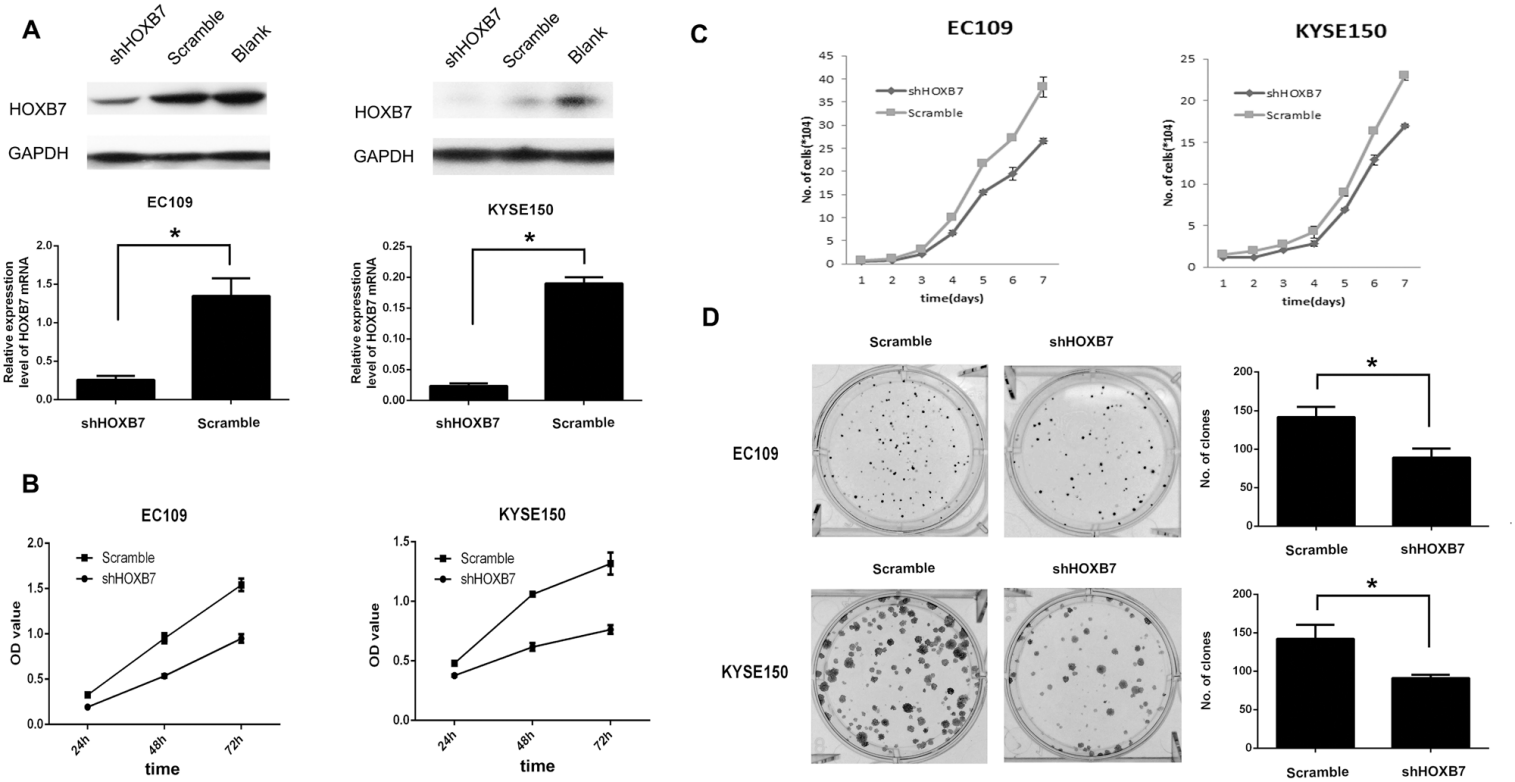
HOXB7 promotes human ESCC cell growth and proliferation. (A) Knockdown of endogenous HOXB7 in specific shRNA-transduced stable EC109 and KYSE150 cells, analyzed by real time PCR and western blotting. (B) Knockdown of HOXB7 inhibits cell growth as determined by CCK8 assay. (C) Knockdown of HOXB7 inhibits cell growth as confirmed by manual cell counts. (D) Knockdown of HOXB7 inhibits cell growth as showed by colony formation assay. Error bars represent mean±SD from 3 independent experiments. *, P<0.05.

To evaluate the possible role of HOXB7 in the proliferation of human ESCC cells, we firstly detected the expression of HOXB7 in several ESCC cell lines. Real-time PCR and western blotting analysis revealed that EC109 and KYSE150 exhibited relatively high endogenous expression of HOXB7. Therefore, we chose EC109 and KYSE150 cell lines for study and knocked down HOXB7 expression by using RNA interference. Of the four lentivirus with short hairpin RNA, the best one was selected to established cell strain with stable knockdown of HOXB7 protein. Meanwhile, one lentivirus with scramble shRNA was used as a control. Finally, EC109/Scramble, EC109/shHOXB7 and KYSE150/Scramble, KYSE150/shHOXB7 were established. The efficacy of knockdown was examined by real-time PCR and Western blotting. HOXB7 mRNA and protein expression was effectively reduced by 80% to 85% in EC109 and KYSE150 cell lines (Fig 2A). CCK8 assay showed that HOXB7 knockdown decreased the proliferation of EC109 and KYSE150 compared to the control cells (Fig 2B; P <0.05). Manual cell counts of cells confirmed the decrease of proliferation seen in the CCK8 assay. (Fig 2C; P<0.05). Colony formation assay revealed that EC109/shHOXB7 and KYSE150/shHOXB7 cells formed much less and smaller colonies than that of control cells (Fig 2D; P < 0.05).

### Down-regulation of HOXB7 protein decreased tumor growth in vivo

**Fig 3:**
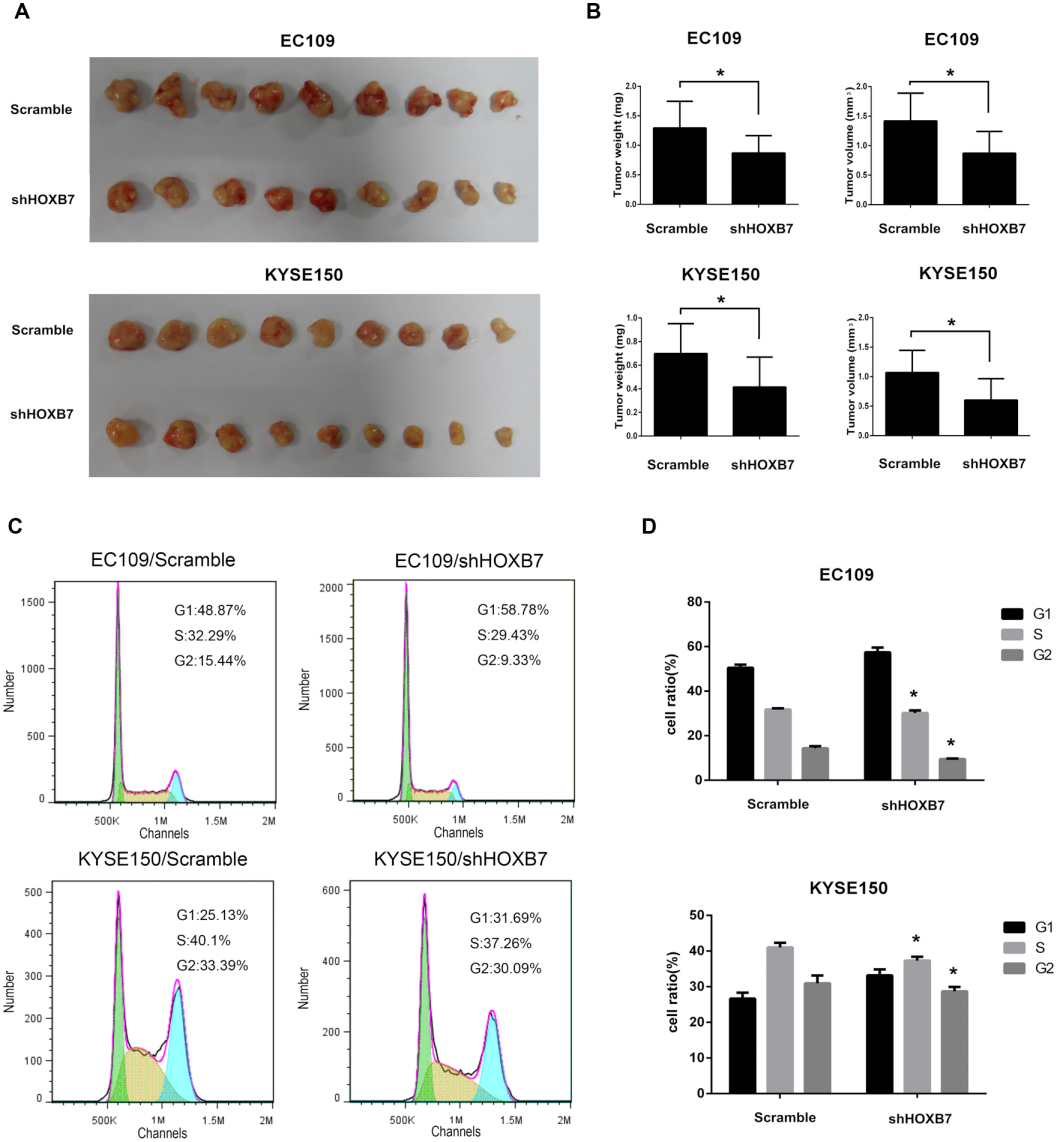
Down-regulated HOXB7 decreased cell proliferation in vivo and its effect on cell cycle distribution. (A) Tumors generated by injection of EC109/KYSE150 with stable knockdown HOXB7 protein and control cells. (B) The weight and volume of tumors from the HOXB7-knockdown cells were relatively lower than those from the control cells. (C) Representative histograms analyzed by flow cytometry showed the cell cycle profiles of the ESCC cells. In the cells with stable knockdown HOXB7 protein, the cell number at S and G2 phases decreased compared with the control cells. (D) Proportion of cells in different phases of the cell cycle. Error bars represent mean±SD from 3 independent experiments. *, P<0.05.

To confirm the effect of HOXB7 on the tumorigenic activity of ESCC cells in vivo, we performed tumorigenesis assays in nude mice. Cell strains with EC109/Scramble, EC109/shHOXB7 and KYSE150/Scramble, KYSE150/shHOXB7 cells were injected into nude mice. The Xenograft tumors were removed 4 weeks after injection and were measured and weighted. As shown in Fig 3A and 3B, the tumors formed by the EC109/shHOXB7 and KYSE150/shHOXB7 cells were significantly smaller than those of control cells-formed tumors. The average weight and volume of tumors in EC109/shHOXB7 group were 869±295 mg and 870±370 mm^3^, respectively, compared with 1292±453 mg and 1416±472 mm^3^ in the EC109/Scramble group (P <0.05). The average weight and volume of tumors in KYSE150/shHOXB7 group were 415±254 mg and 603±362 mm^3^, respectively, compared with 697±253 mg and 1069±377 mm^3^ in the KYSE150/Scramble group (P <0.05).

### HOXB7 accelerated cell cycle progression

To explore the possible mechanism of HOXB7 in promoting ESCC cell proliferation, we tested the cell cycle distribution by flow cytometry. As shown in Fig 3C, knockdown of HOXB7 protein resulted in G1 arrest, with an increase in G1 proportion and a decrease in S and G2 proportion. The percentage of cells in S and G2 phases in EC109/shHOXB7 group was 30.18%±1.16% and 9.51%±0.23% respectively, which was lower than that in control cells (31.75%±0.55% and 14.36%±0.94%, Fig 3D, P<0.05). The average percentage of cells in S and G2 phases in KYSE150/shHOXB7 group was 37.31%±1.09% and 28.70%±1.22% respectively, compared with 41.00%±1.32% and 30.92%±2.21% in the KYSE150/Scramble group (Fig 3D, P<0.05). These results indicated that HOXB7 might accelerate the G1 to S-phase transition in ESCC cell lines, which contributes to ESCC cell proliferation.

## Discussion

Ideal tumor biomarkers have good prognostic value and can guide clinical practice, and therefore, the search for a valuable prognostic biomarker has gained prime focus in oncology research. Among these, abnormal expression of embryogenesis-related genes in malignancy has always received significant attention [10]. For example, the relationship between carcinoembryonic antigen (CEA) and colorectal cancer and that between alpha-fetoprotein (AFP) and liver cancer, which have been successfully applied in clinical settings. However, until now, good tumor markers for ESCC have not been identified for use in clinical practice. Our previous studies have revealed abnormal expression of 39 members of HOX genes at the mRNA level in malignant esophagus tissue. Among them, eight were expressed only in the malignant tissue from ESCC patients, but not in the paired adjacent normal tissues, including HOXB7 [9]. Other study also showed that HOXB7 is not expressed in the normal esophagus but is expressed at higher levels at the mRNA level in ESCC [11]. The present study confirmed these preliminary findings. It was showed that the expression of HOXB7 was significantly higher in 23 samples of malignant esophageal tissue than in the paired adjacent normal mucosa, which offers a good basis to consider HOXB7 as a potential molecular marker for ESCC. Since Jan. 2000, we have been establishing a single-surgeon (Dr. Chen) prospective esophageal cancer database and consecutively recruited 1,249 patients with esophageal cancer who underwent surgical resection. Follow-up visits were scheduled for all patients included in the database until their death. HOXB7 expression in the extracted 177 cases that met the criteria was examined, and its relationship with prognosis was analyzed. The results showed that HOXB7 expression was positively correlated with the TNM stage (P = 0.034), T stage (P = 0.036), lymph node metastasis (P = 0.042). The median survival time of patients with high HOXB7 expression was significantly shorter than that of patients with low HOXB7 expression (45 months vs. 137 months, p = 0.007). To further confirm this result, we conducted a retrospective study on another previous cohort of patients with no prospective data. A similar trend was noted in our results: HOXB7 expression in this cohort also showed a positive correlation with the TNM stage (P = 0.001), T stage (P = 0.000) and lymph node metastasis (P = 0.006). The median survival time of patients with high HOXB7 expression was significantly shorter than that of patients with low HOXB7 expression (19 months vs. 34 months, p = 0.001). Moreover, multivariate analysis with variables including age, sex, tumor location, TNM stage, T stage, N stage, and HOXB7 expression showed that HOXB7 expression and TNM stage were independent prognostic factors in 177 patients recruited in the prospective database (HR[95%CI] = 0.573, [0.341–0.963], p = 0.036) and 103 patients recruited in another cohort without prospective data (HR[95%CI] = 0.543, [0.350–0.844], p = 0.024), indicating that HOXB7 had prognostic value for patients with ESCC.

To further explore the functional mechanism of HOXB7 in the occurrence and development of ESCC, we examined HOXB7 expression in several ESCC cell lines and shortlisted two cell lines showing high expression of HOXB7 (EC109 and KYSE150) for in vitro and in vivo studies.

First, RNA interference technique was employed to establish stable HOXB7-knockdown cell strains, which were then used to carry out CCK8 assay, colony-forming rate assay, and cell cycle analysis by flow cytometry to observe the effect of HOXB7 on cell proliferation in ESCC. Our results showed that the rate of cell proliferation was slower in HOXB7-knockdown cell strains, colony formation rate was reduced, G1-phase arrest occurred in the malignant cells, and the proportion of cells in G1 phases increased significantly. To further verify the impact of HOXB7 on in vivo cell proliferation, we used knockdown cell strains to establish tumorigenicity in a nude mice model and found that the tumorigenic capacity of knockdown cell strains was significantly lower than that of control cells.

In fact, the dysregulation of HOXB7 expression has been reported in a variety of tumors, including breast cancer [12–14], ovarian cancer [15], oral cancer [16], colorectal cancer [17], lung cancer [18], melanoma [19–21] and pancreatic cancer [22–24]. The overexpression of HOXB7 was showed to be related to poor prognosis in breast cancer [14], oral cancer [16], colorectal cancer [17], lung cancer [18] and pancreatic adenocarcinoma [22, 23]. But the clinical significance of HOXB7 in ESCC are rarely reported. There were two articles exploring prognostic significance of HOXB7 expression at mRNA and protein level in ESCC recently, but the sample sizes were very limited, and they did not confirm their findings in an independent cohort [25, 26]. Compared to these studies, we not only verified the reliability of our findings in two independent cohorts including 280 cases, but also studied further to explore the possible function of HOXB7 in ESCC. It has been found that HOXB7 could promote tumor cell proliferation in other solid tumors [13–19, 27]. But until now there were no studies exploring the possible role of HOXB7 and its underlying mechanisms in ESCC. Based on the above-discussed preliminary findings, we believe that HOXB7 is likely to be identified as a prognostic biomarker for ESCC patients, and abnormal HOXB7 expression could affect the proliferative capacity of cells.

Meanwhile, we noted a positive correlation between higher HOXB7 protein expression and regional lymph node metastasis. So we conducted Transwell migration assay and Matrigel invasion assay, which indicated that HOXB7 knockdown inhibited cell migration and invasion ability of EC109 and KYSE150 (S1 Fig; P < 0.05). But the function of HOXB7 in cell invasion and metastasis in ESCC still need further exploration.

The mechanisms that underlie the role of HOXB7 in ESCC is unclear. The exploration of mechanism is complicated by the sequence similarity among HOX genes and functional redundancy recognized in the HOX gene system [7]. As a transcriptional factor, HOXB7 mediates its effects by regulating the transcription of target genes. Although many genes have been shown to be directly or indirectly regulated by HOXB7 in other cancer cells, only a few of them have been identified as direct targets, including bFGF [13, 22, 28]. HOXB7 could directly activate the expression of basic fibroblast growth factor (bFGF) and promote growth of various tumors, such as breast cancer and melanoma [13, 19, 29]. And bFGF had been implicated in diverse biological processes, such as proliferation, differentiation, survival, and migration [30]. HOXB7 expression could promote cell growth by triggering both intracrine and autocrine bFGF growth stimulatory pathways in ovarian carcinomas [15]. The expression of bFGF could activate Ras-RAF-MAPK pathway through autocrine signaling cascades in breast cancer [12]. It was showed that PI3K/AKT and MAPK pathways were activated by HOXB7 in colorectal cancer, which may explain the accelerating G1–S transition induced by HOXB7 [17]. In conclusion, these results reinforced our findings and clarified the importance of HOXB7 in tumorigenesis. Further studies to identify mechanisms about HOXB7 upstream activators, downstream targets and functional partners would be worthwhile to better understand the role of HOXB7 in ESCC.

This study has some limitations: the clinical data were obtained from the single center, the sample size was not large enough, and clinical research studies were retrospective actually. Therefore, in future studies, the sample size should be increased, and patients from multiple center should be included to clarify whether HOXB7 can serve as a specific molecular prognostic marker in ESCC. Furthermore, an in-depth study on the molecular mechanisms that underlie the role of HOXB7 in promoting the proliferation of ESCC cells is warranted.

## Supporting Information

S1 Fig HOXB7 promotes human ESCC cell migration and invasion. (A) Knockdown of HOXB7 inhibits cell migration as determined by Transwell migration assay. (B) Knockdown of HOXB7 inhibits cell growth as showed by Matrigel invasion assay. Error bars represent mean±SD from 3 independent experiments. *, P<0.05.
(TIF)

S1 File Supplemental Materials and Methods. Description of Migration and Invasion Assays.
(DOCX)
