# Supplementary figures and images for: Deregulated HOXB7 Expression Predicts Poor Prognosis of Patients with Esophageal Squamous Cell Carcinoma and Regulates Cancer Cell Proliferation In Vitro and In Vivo
Source: PLoS One. 2015 Jun 15;10(6):e0130551. doi: 10.1371/journal.pone.0130551 (PMC4468077; doi:10.1371/journal.pone.0130551)

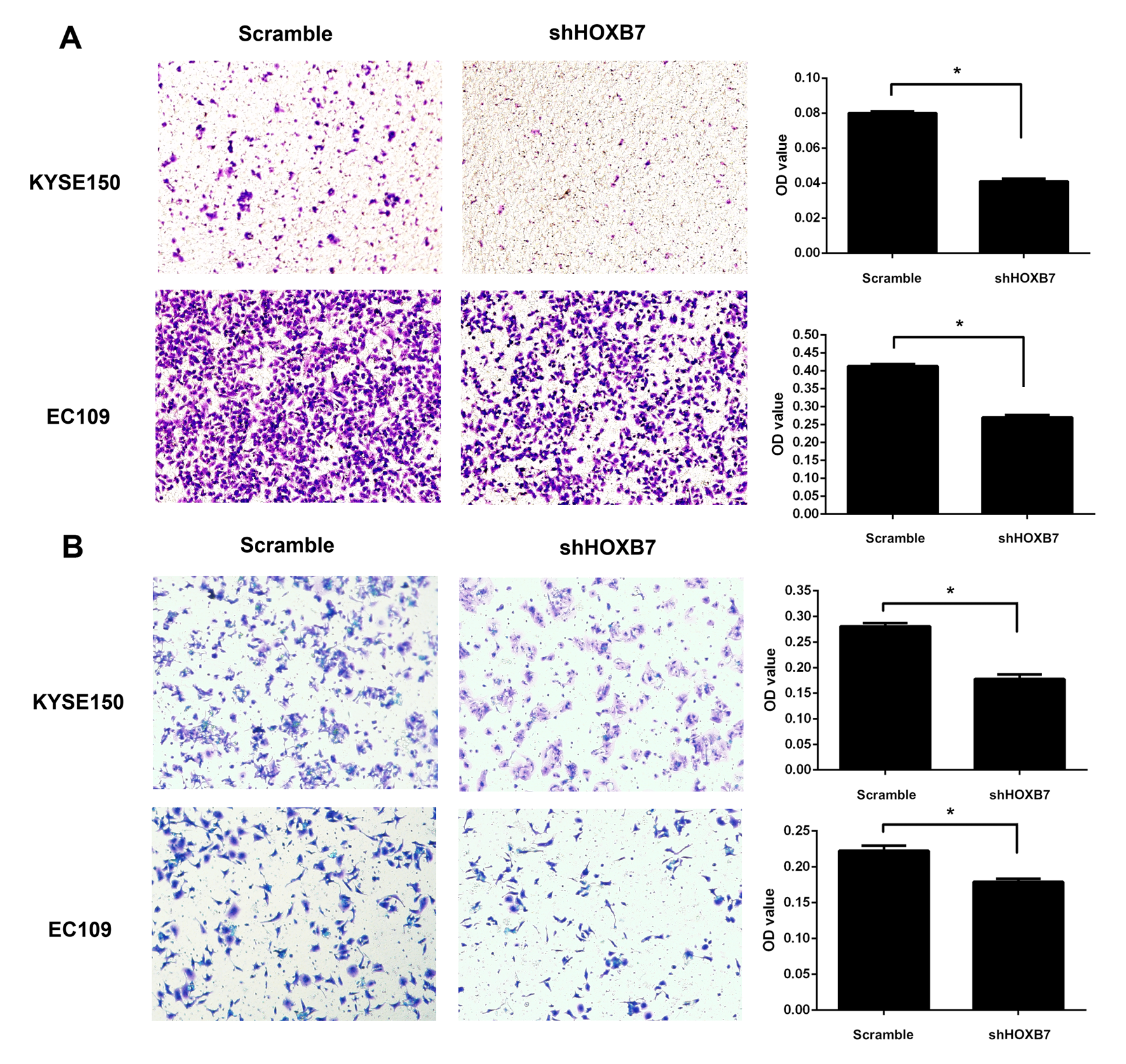

Supplement: S1 Fig — (A) Knockdown of HOXB7 inhibits cell migration as determined by Transwell migration assay. (B) Knockdown of HOXB7 inhibits cell growth as showed by Matrigel invasion assay. Error bars represent mean±SD from 3 independent experiments. *, P<0.05. (TIF) [file pone.0130551.s001.tif]
