# Supplementary material for: Deregulated HOXB7 Expression Predicts Poor Prognosis of Patients with Esophageal Squamous Cell Carcinoma and Regulates Cancer Cell Proliferation In Vitro and In Vivo
Source: PLoS One. 2015 Jun 15;10(6):e0130551. doi: 10.1371/journal.pone.0130551 (PMC4468077; doi:10.1371/journal.pone.0130551)
Supplement: S1 File — Description of Migration and Invasion Assays. (DOCX) [file pone.0130551.s002.docx]

**Supporting Information**

**Deregulated HOXB7 expression predicts poor prognosis of patients with esophageal squamous cell carcinoma and regulates cancer cell proliferation in vitro and in vivo**

**Hui Li^1^, Lu-Yan Shen^1^, Wan-Pu Yan^1^, Bin Dong^2^, Xiao-Zheng Kang^1^, Liang Dai^1^, Yong-Bo Yang^1^, Hao Fu^1^, He-Li Yang^1^, Hai-Tao Zhou^1^, Chuan Huang^1^, Zhen Liang^1^, Hong-Chao Xiong^1^, and Ke-Neng Chen^1*^**

**1** Department of Thoracic Surgery I, Key Laboratory of Carcinogenesis and Translational Research (Ministry of Education), Beijing Cancer Hospital, Peking University School of Oncology, Beijing, People’s Republic of China

**2** Department of Pathology, Beijing Cancer Hospital, Peking University School of Oncology, Beijing, People’s Republic of China

* Corresponding author

E-mail: chenkeneng@bjmu.edu.cn (KNC)

S1_File. Supplemental Materials and Methods

Migration and Invasion Assays

Tumor cell invasion was assessed using BD Biocoat Matrigel Invasion Chambers with 8μm porous membranes (BD Biosciences). Cells (1 ×10^5^) were seeded in the upper chambers in serum-free medium. Medium with 10% FBS was injected into the lower chamber. After 48 hours incubation, cells were removed from the upper chambers and cells on the bottom side were fixed, stained with 0.1% crystal violet solution and destained in 33% acetic acid. The numbers of invaded cells were evaluated by detecting the absorbance of acetic acid, measured at 570 nm. Tumor cell migration assay were performed following the same method using uncoated membranes with 24 hours incubation.
